# Supplementary material for: Allosteric enhancement of ORP1-mediated cholesterol transport by PI(4,5)P2/PI(3,4)P2
Source: Nat Commun. 2019 Feb 19;10:829. doi: 10.1038/s41467-019-08791-0 (PMC6381110; doi:10.1038/s41467-019-08791-0)
Supplement: Supplementary file 1 — Supplementary Information [file 41467_2019_8791_MOESM1_ESM.pdf]

Supplementary Information

Allosteric enhancement of ORP1-mediated cholesterol transport by $PI(4,5)P_2$/$PI(3,4)P_2$

Dong et al.

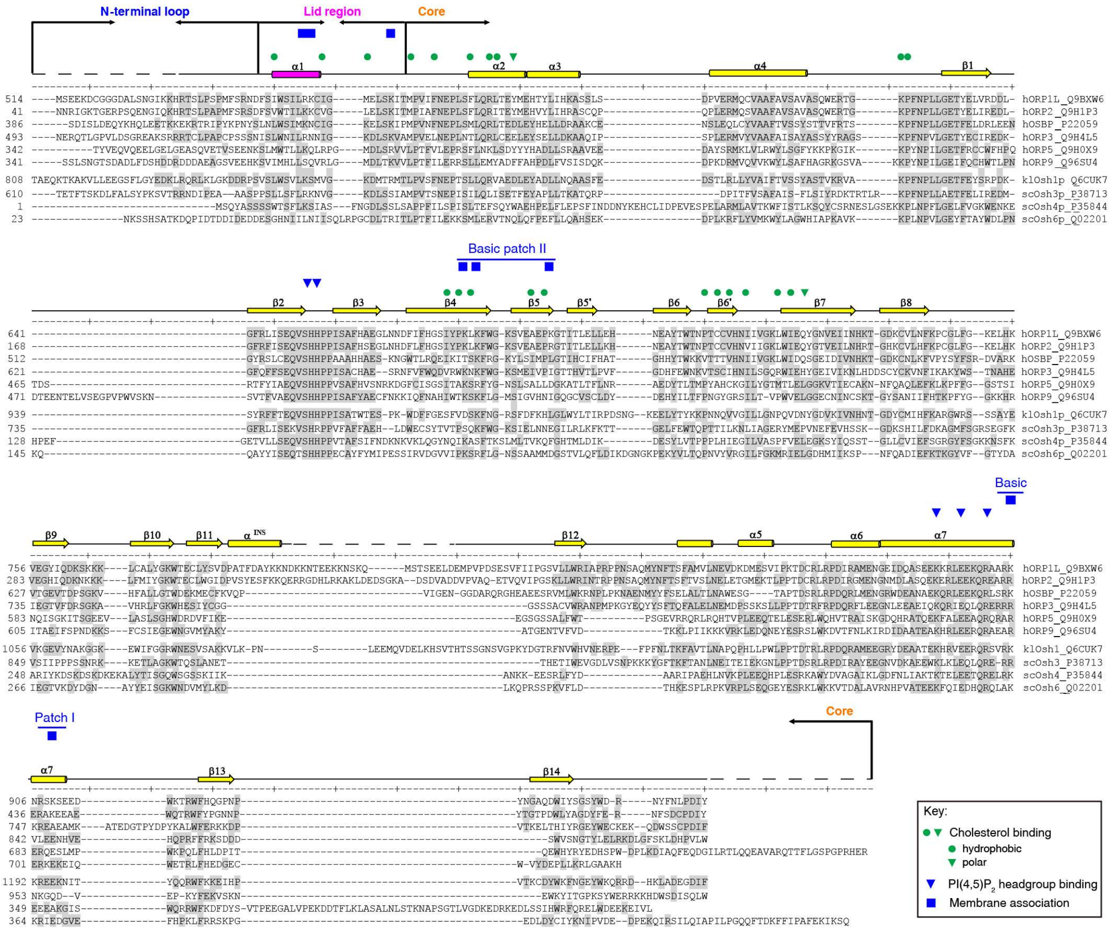

Supplementary Fig. 1 Structure-based sequence alignment of the ORDs from ORP/Osh proteins. Residues of ORP1-ORD participating in the binding of cholesterol, PI(4,5)P_2 headgroup and membrane association are respectively indicated. The code following each protein name is the corresponding UniProt ID. h, human; sc, *Saccharomyces cerevisiae*; kl, *Kluyveromyces lactis*.

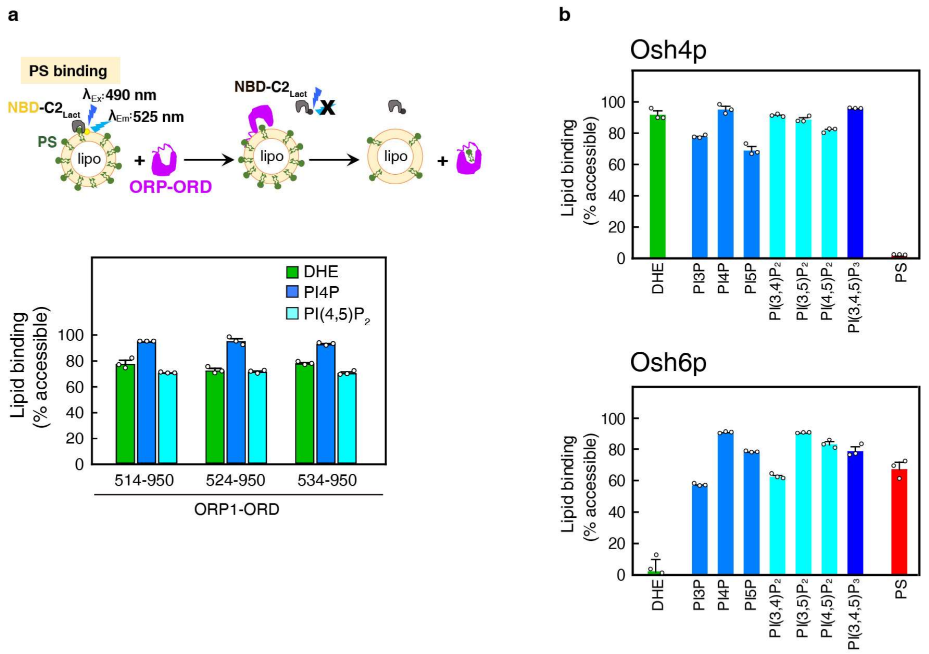

Supplementary Fig. 2 In vitro binding analyses of ORP/Osh family members. a, Lipid binding assays of three ORP1-ORD fragments. Shown at the top is the scheme of the PS binding assay. The three soluble ORP1-ORD proteins had no apparent difference in their lipid binding abilities, and the shortest one (residues 534-950) was used in following biochemical assays unless indicated. **b,** Lipid binding analyses for yeast Osh4p and Osh6p. (In all bar graphs, data are shown as mean ± s.e.m.(error bar), *n* = 3)

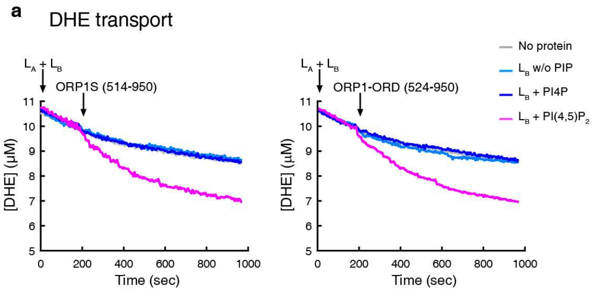

b

	DHE Transfer	v_0 (DHE min^{-1} *per* ORD)
**ORP1**	basal	0.39 ± 0.08
	PI(4,5)P_2-simulated	7.02 ± 0.49
**Osh4p**	basal	2 ± 0.1
	PI4P-simulated	17.4 ± 1.1

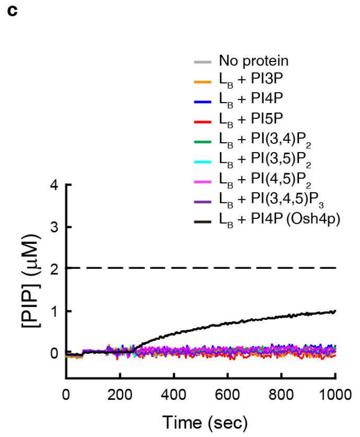

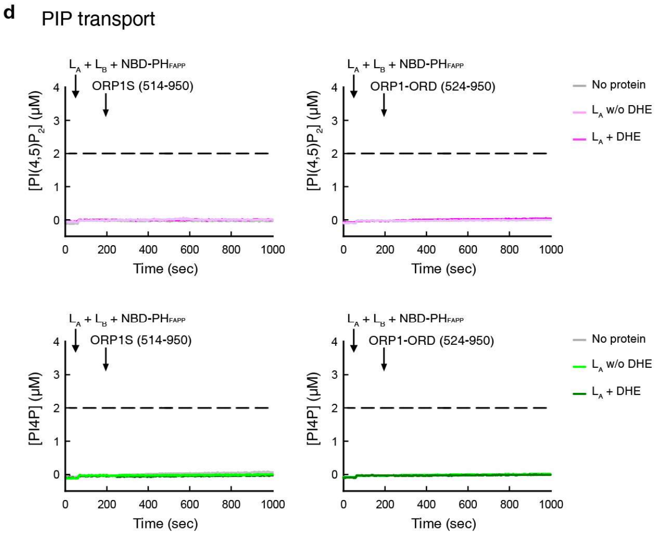

Supplementary Fig. 3 In vitro transfer analyses of ORP/Osh proteins. a, DHE transport assays of ORP1S (residues 514-950) and ORP1-ORD (524-950) with or without PIP in acceptor liposomes. **b**, The basal and PIP-stimulated initial velocities of DHE transport by ORP1-ORD and Osh4p. (Data are shown as mean ± s.e.m., $n = 3$) **c**, PIP transport assays of ORP1-ORD without DHE in acceptor liposomes. PI4P transport by Osh4p in the absence of DHE was shown as control. **d,** PI(4,5)P_2 (top) and PI4P (bottom) transport assays of ORP1S (residues 514-950) and ORP1-ORD (524-950) with or without DHE in acceptor liposomes.

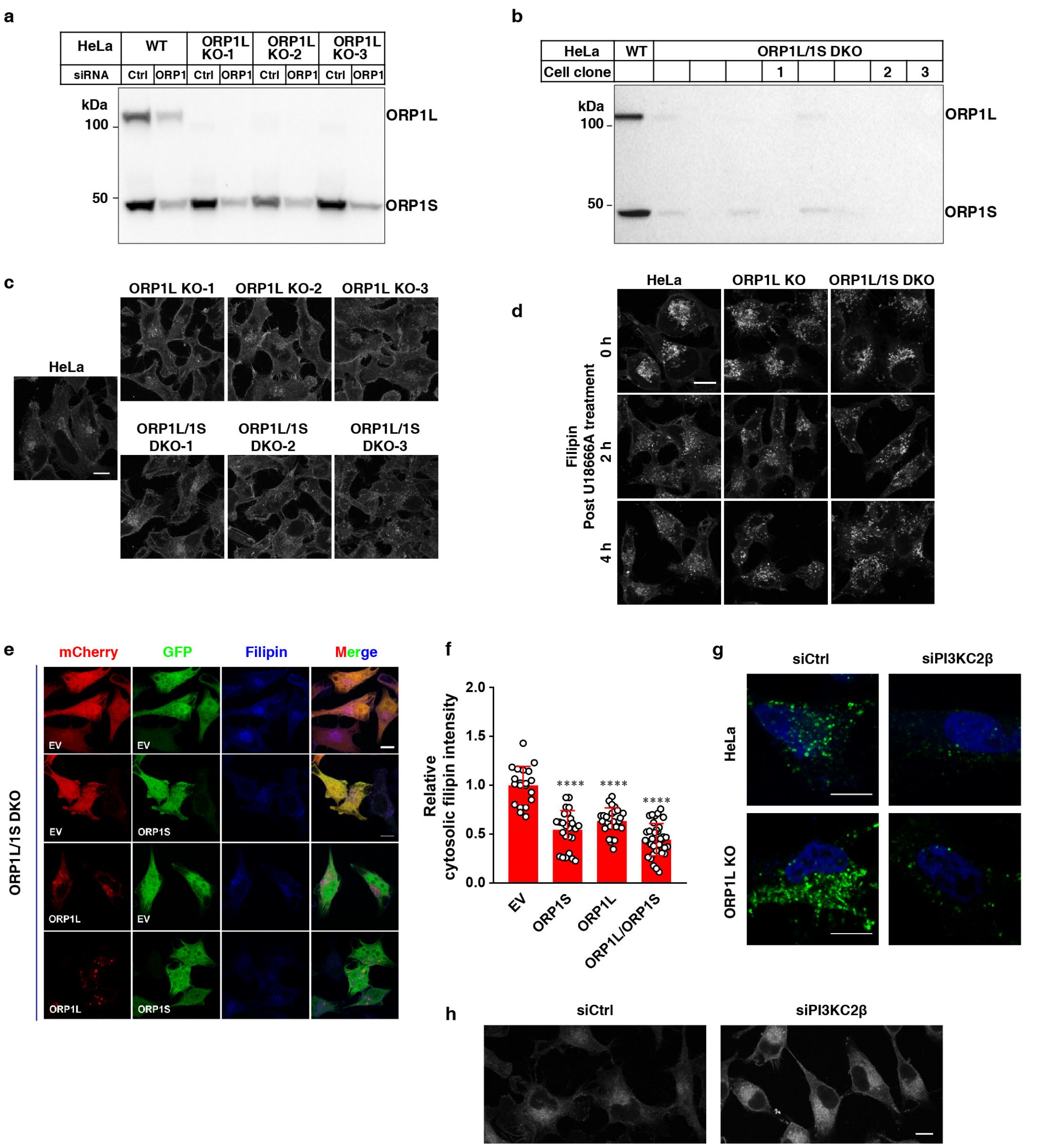

Supplementary Fig. 4 Involvement of ORP1 in endosomal cholesterol transport and the effects of PIPs in vivo. a, Western blotting analyses of HeLa and ORP1L KO cells treated with control siRNA or siORP1. **b,** Western blotting analyses of HeLa and ORP1L/ORP1S DKO cells. **c,** Filipin staining of HeLa control cells and the ORP1L KO, ORP1L/1S DKO clones. (Bar = 10 μm) **d,** U18666A-meidated LEL cholesterol release assays in ORP1L KO, ORP1L/1S DKO and HeLa control cells. (Bar = 10 μm) **e**, Filipin staining of ORP1L/1S DKO cells expressing empty vector (EV), mCherry-ORP1L and/or GFP-ORP1S. (Bars = 10 μm) **f**, Quantitation of cytosolic filipin intensities of cells in panel e. (****, $p < 0.0001$; n=20-36) Statistical analyses use one-way ANOVA. **g**, Immunostaining of intracellular PI(3,4)P_2 in HeLa and ORP1L KO cells treated with control or PI3KC2β siRNAs. (Bars = 10 μm) **h**, U18666A-mediated LEL cholesterol release assays in HeLa cells treated with control siRNAs or siPI3KC2β. (Bars = 10 μm)

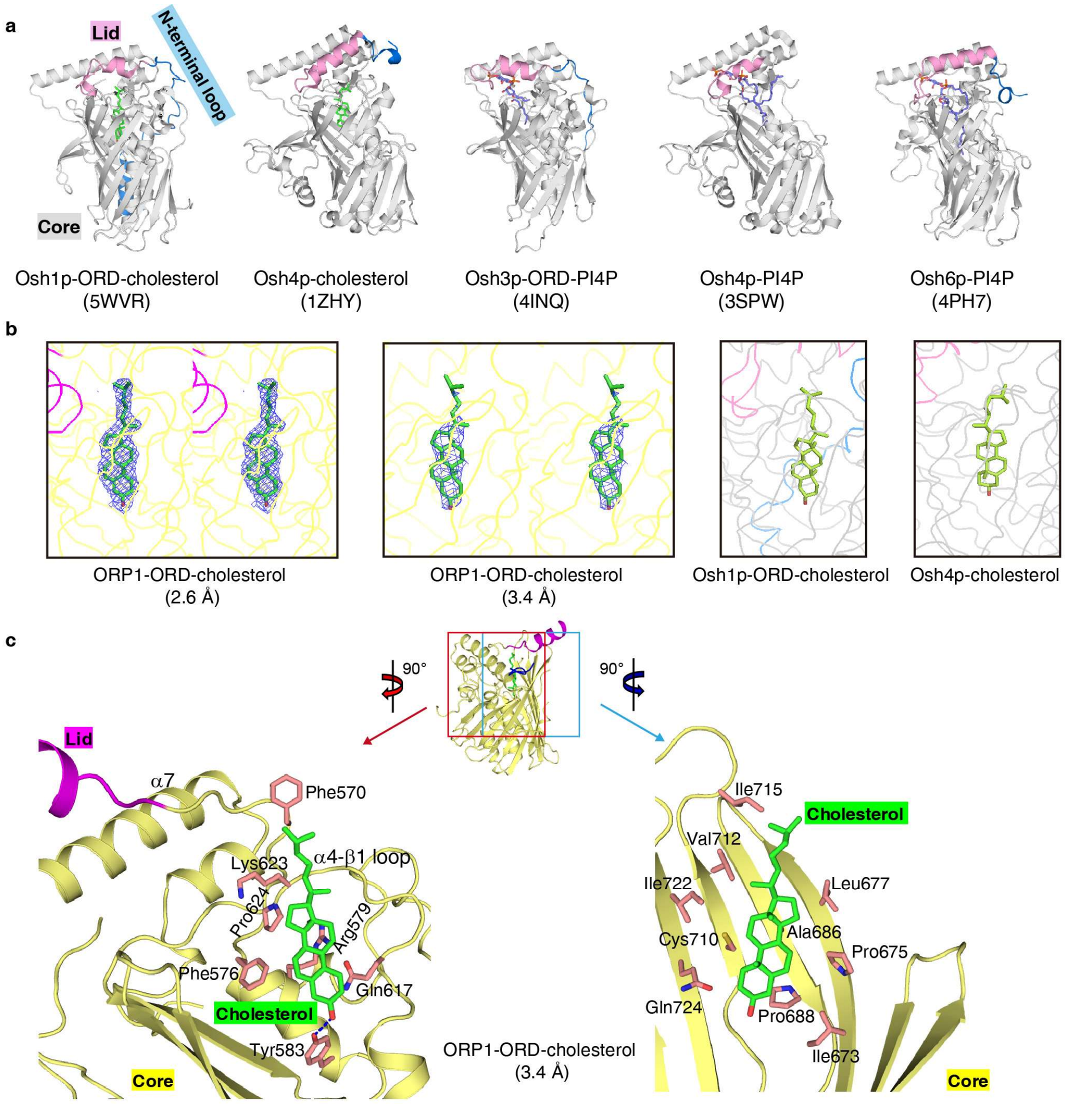

Supplementary Fig. 5 Binding mode of cholesterol in ORP1-ORD. a, Structures of yeast Osh proteins in complex with cholesterol or PI4P in closed conformations. The structure core of each yeast ORD is shown in grey in the same orientation as in Fig. 4a, b. The N-terminal loop and lid region are highlighted in marine blue and pink, respectively. **b**, Comparison of cholesterol binding modes in ORP1-ORD structures and yeast Osh proteins. The stereo images of 2Fo-Fc omit maps (contoured at 1.0 σ) revealed reasonable electron density for the cholesterol ligands in both ORP1-ORD-cholesterol complex structures. **c,** Cholesterol binding mode in the 3.4 Å ORP1-ORD-cholesterol complex structure. The binding mode is highly conserved, except that the lid region was not involved in ligand binding.

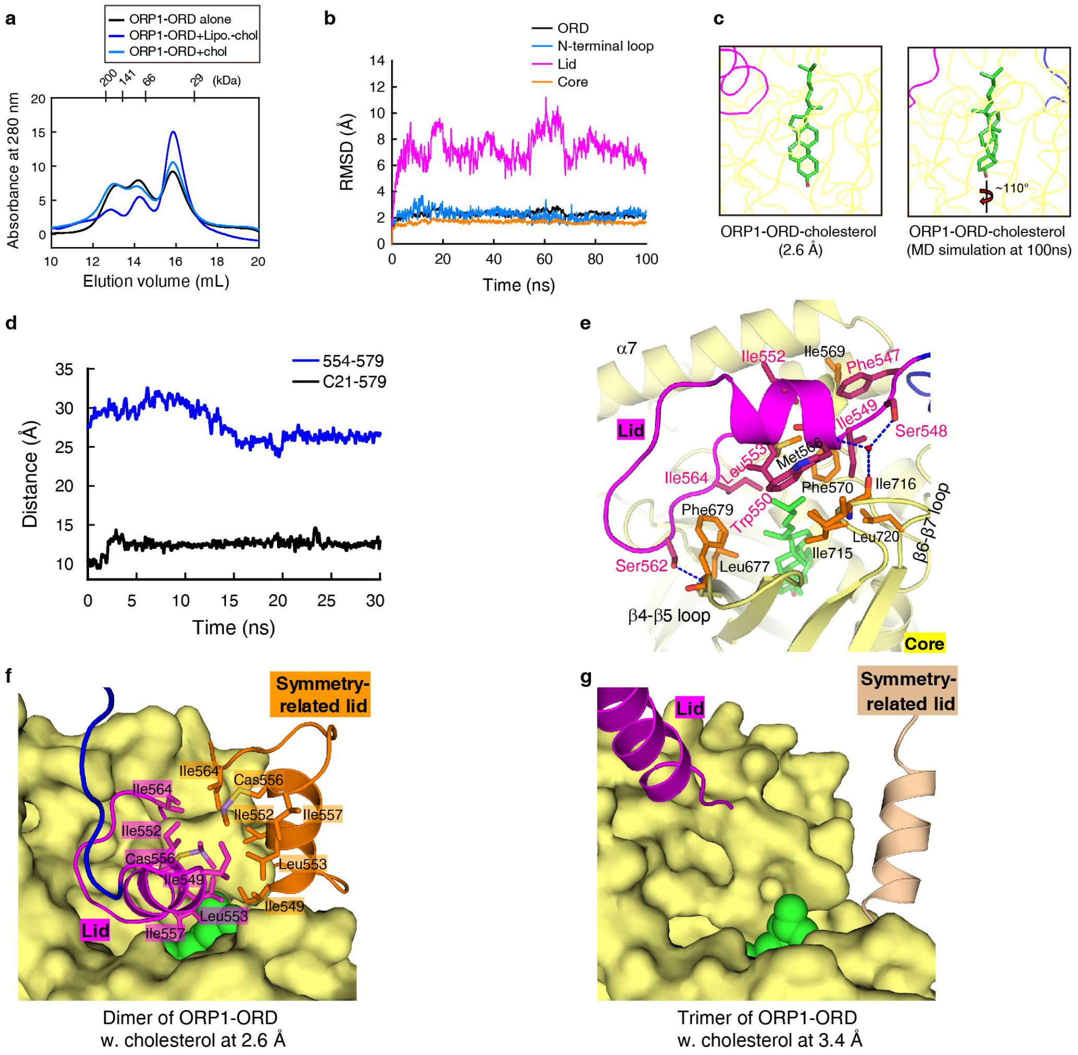

Supplementary Fig. 6 The lid can seal the lipid binding tunnel in different oligomeric states. a, Gel filtration analyses of ORP1-ORD (534-950) upon cholesterol binding. **b**, Backbone RMSD values of different regions in ORP1-ORD as a function of MD simulation time. **c**, Comparison of cholesterol configurations in the ORP1-ORD structure and final MD model. **d,** Comparison of the cholesterol rotation and lid closing movements in the first 30 ns simulation. Movement of the lid was quantified by measuring the distance between the Cα of Arg554 from the lid and the Cα of Arg579 in the core. **e**, Close-up view of the detailed interactions between the closed lid and the core in the 100 ns MD model. **f, g,** The lid regions can obstruct the binding tunnels in the cholesterol-bound ORP1-ORD dimer (f) and trimer (g).

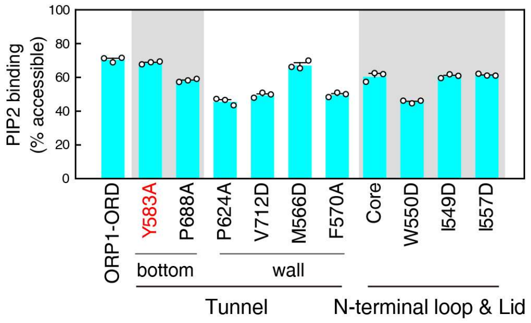

Supplementary Fig. 7 PI(4,5)P_2 binding analyses for ORP1-ORD mutations at the cholesterol binding tunnel and the N-terminal loop and lid region. (Data are shown as mean ± s.e.m. (error bar), n = 3)

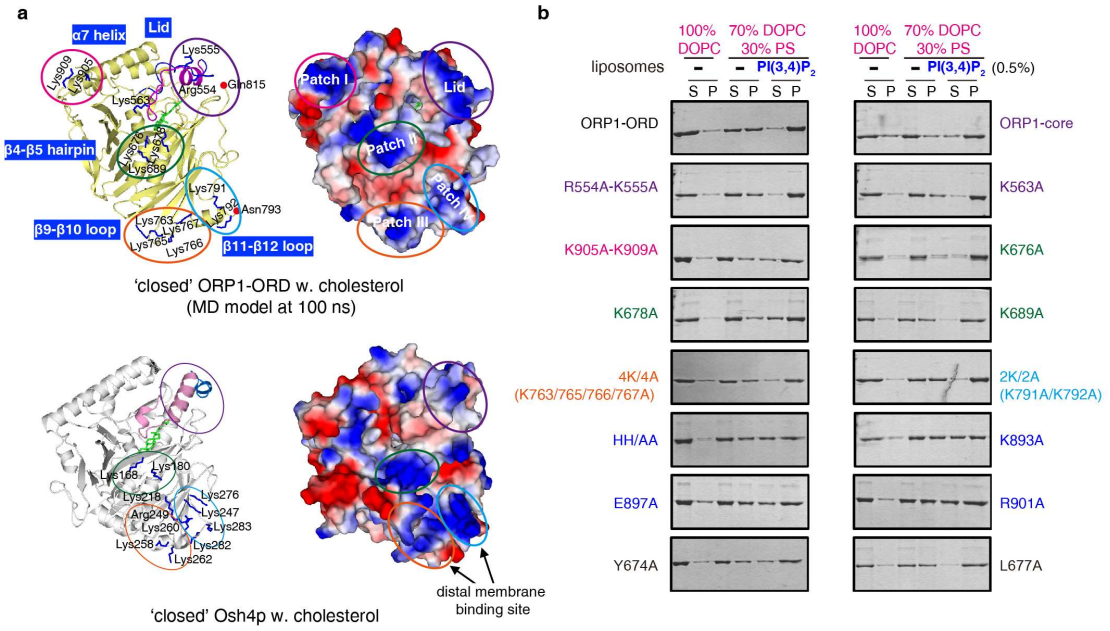

Supplementary Fig. 8 Membrane targeting of ORP1-ORD. a, Potential membrane binding sites on structures of the closed cholesterol-bound ORP1-ORD (MD model) and the closed cholesterol-bound Osh4p. The surface representations are colored according to electrostatic potential (positive, blue; negative, red), and the lid region and basic patches are circled. **b,** Liposome association assays for ORP1-ORD mutations of the lid region, four basic patches, the polar interface of PI head group binding and two hydrophobic residues at the tunnel entrance. Differences in the liposome constitutions were indicated at the top. The supernatant (S) and pellet (P) fractions were analyzed by SDS-PAGE and quantified by Image-J.

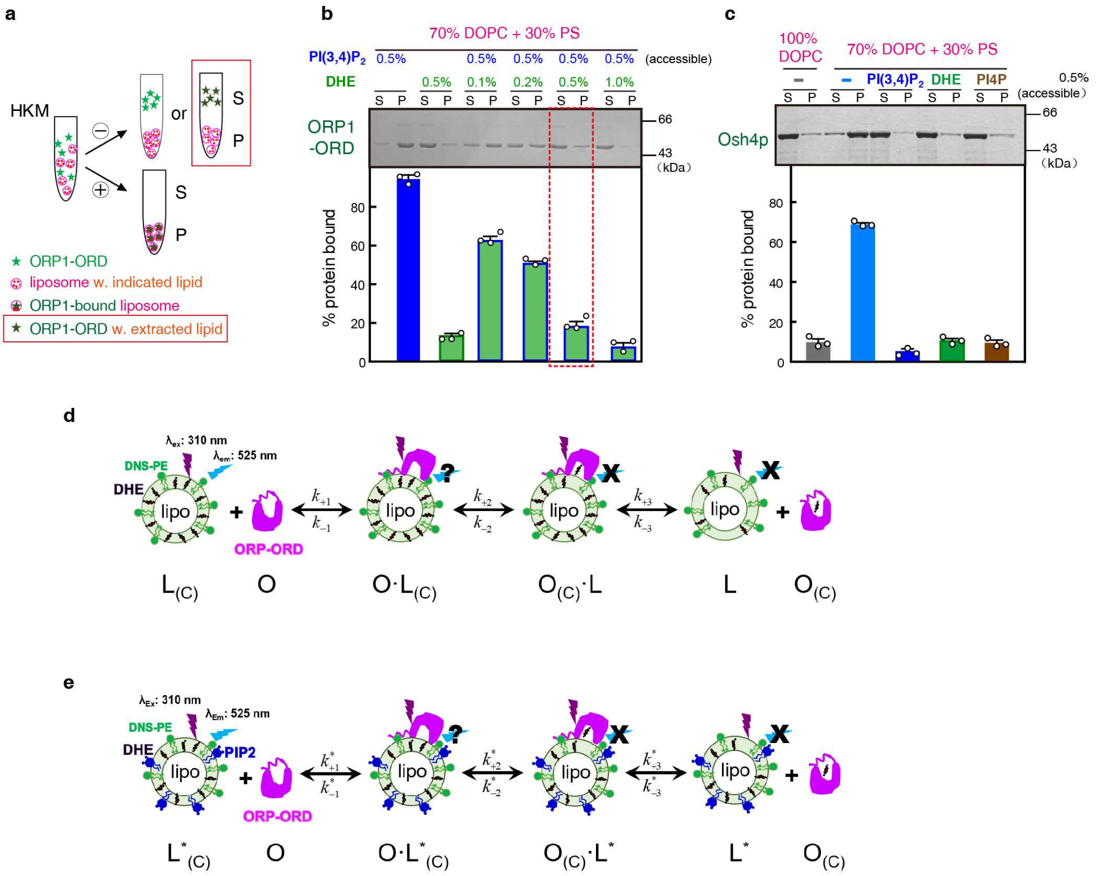

Supplementary Fig. 9 Membrane targeting of Osh4p. a, Schematic diagram of the modified liposome association assay. In addition to DOPC/PS, the liposomes were doped with 0.5% indicated lipid. **b,** Membrane association assays of ORP1-ORD using liposomes doped with 0.5% PI(3,4)P_2 and increasing amount (0.1-1.0%) of DHE. The data shown in Fig. 8a were boxed. **c,** Membrane association analyses of Osh4p. Osh4p can bind to membrane through electrostatic interactions with acidic phospholipids, such as PS; on the other hand, cholesterol and PIPs can be extracted from the liposome/membrane by Osh4p. (In the bar graphs, data are shown as mean ± s.e.m. (error bar), n = 3) **d, e,** cholesterol binding and extraction by ORP1-ORD without (d) or with (e) PI(3,4)P_2 stimulation. $L_{(C)}$ and L correspond to the cholesterol/DHE-doped and cholesterol-extracted liposomes. O and $O_{(C)}$ correspond to apo-ORP1 and cholesterol-bound ORP1 in solution. $O \bullet L_{(C)}$ is the binary complex formed between O and $L_{(C)}$, and $O_{(C)} \bullet L$ is the binary complex of $O_{(C)}$ and L.

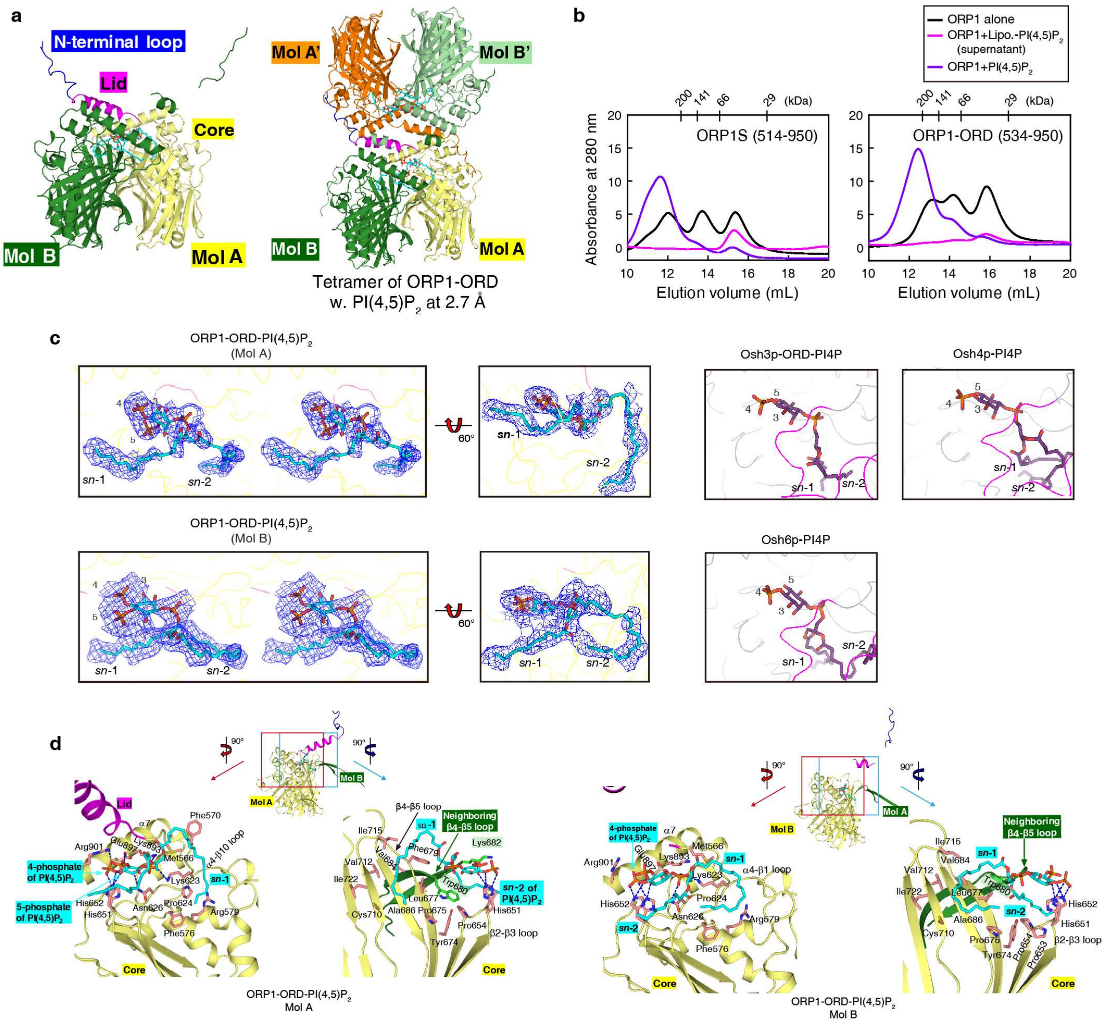

Supplementary Fig. 10 Structure of PI(4,5)P₂-bound ORP1-ORD. **a**, Two ORP1-ORD-PI(4,5)P_2 complexes within an asymmetric unit. Mol A is colored the same as in Fig. 8d, and Mol B is shown in dark-green. The crystals were obtained using the direct mixture of ORP1-ORD and free PI(4,5)P_2, where the ORP1-ORD-PI(4,5)P_2 complex forms tetramer through domain-swapping interactions between symmetry-related molecules. **b**, Gel filtration analyses of two ORP1-ORD proteins upon PI(4,5)P_2 binding. ORP1-ORD alone, direct mixture of protein and free PI(4,5)P_2, and supernatant of the protein and PI(4,5)P_2-containing liposome mixture (centrifuged at 400,000 ×g for 1 hr at 4°C) were analyzed. The shift of ORP1S (514-950) may be due to the presence of 20 flexible amino acids at the N-terminus. The direct mixture of ORP1-ORD and free PI(4,5)P_2 behaves as tetramers in solution, and indeed forms tetramers in crystals. **c**, Comparison of the PI(4,5)P_2 and PI4P binding modes in ORP1 and Osh ORD structures. The Fo-Fc omit maps (contoured at 2.3 σ) revealed reasonable electron density that allowed the fitting of two PI(4,5)P_2 ligands (18:0/20:4, the dominant species of brain mixture): 18 carbon-long *sn*-1 chains in both Mols A and B, and 15 carbon-long or 10 carbon-long *sn*-2 chain in Mol A or Mol B. Stereo images are shown on the left. **d,** PI(4,5)P_2 binding sites in the ORP1-ORD-PI(4,5)P_2 complexes. Only the *sn*-1 stearate chain of each PI(4,5)P_2 ligand sticks into the lipid-binding tunnel, and mainly taking the place of the hydrocarbon tail of cholesterol, while the *sn*-2 arachidonate chain leans out in the opposite direction, interacting with residues from the β2-β3 and β4-β5 loops (including Tyr674 and Leu677). In addition, the *sn*-2 chain of PI(4,5)P_2 bound in one ORP1-ORD reaches for the other protein within an asymmetric unit, stabilized by the neighboring β4-β5 loop (^{680}WGK^{682}).

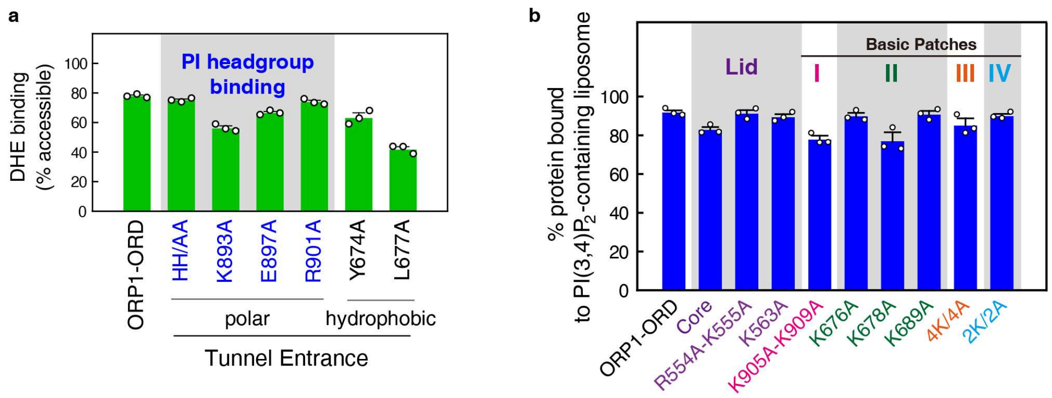

Supplementary Fig. 11 PI headgroup and cholesterol bind to separate sites of ORP1-ORD. a, DHE/cholesterol binding assays of ORP1-ORD mutations at the polar interface indispensable for PI headgroup binding. **b**, Effects of the basic patch mutations on membrane association of ORP1-ORD analyzed using the PI(3,4)P_2-containing liposomes. (In the bar graphs, data are shown as mean ± s.e.m. (error bar), *n* = 3)

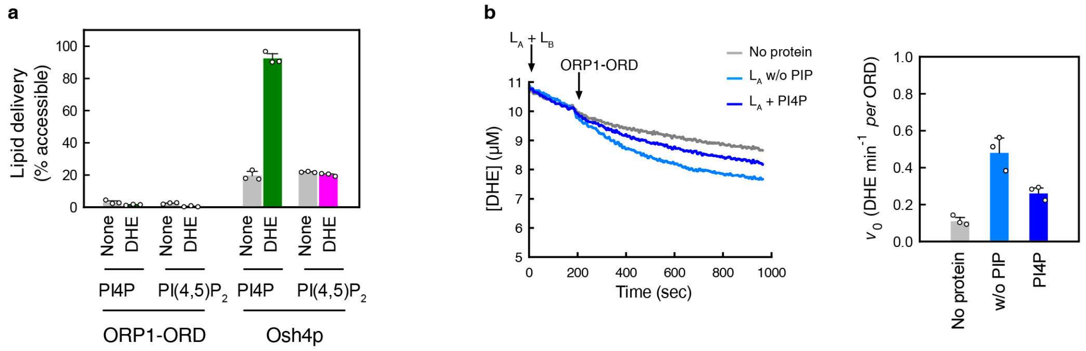

Supplementary Fig. 12 PI4P inhibits DHE transport by ORP1-ORD. a, PIP delivery assays for ORP1-ORD and Osh4p in the absence and presence of DHE/cholesterol. **b,** DHE transport assay of ORP1-ORD with both DHE and PI4P embedded in the donor liposomes. The experiment setup was similar to that in Fig. 3c except that the concentration of ORP1-ORD used here was 0.5 μM. Also shown is the initial velocity of DHE transport by ORP1-ORD. (In the bar graphs, data are shown as mean ± s.e.m. (error bar), *n* = 3)

Supplementary Table 1. Primers for expression constructs.

Primer ID	5'-3' sequence
ORP1-1M-F	GAGCATGTCCATATGATGAACACAGAAGCGGAG
ORP1-514M-F	GAGCATGTCCATATGATGAACACAGAAGCGGAG
ORP1-524D-F	GAGCATGTCCATATGGATGCTCTCTCCAATGGCATCAAG
ORP1-534R-F	GAGCATGTCCATATGATGAGGACAAGTTTGCCTTCT
ORP1-566M-F	GAGCATGTCCATATGATGCCAGTTATATTTAATGAG
ORP1-950Y-R	GTGACGTCACTCGAGATAAATGTCAGGCAAATTGAAG
ORP1L-mCherry-F	GAGCATGTCGAATTCATGAACACAGAAGCGGAG
ORP1L-mCherry-R	GTGACGTCAGTCGACTTAATAAATGTCAGGCAA
ORP1-I549D-F	AGAAATGACTTCAGTGACTGGAGCATCCTCAGA
ORP1-I549D-R	TCTGAGGATGCTCCAGTCACTGAAGTCATTTCT
ORP1-W550D-F	AATGACTTCAGTATCGACAGCATCCTCAGAAAA
ORP1-W550D-R	TTTTCTGAGGATGCTGTCGATACTGAAGTCATT
ORP1-K555A-F	TGGAGCATCCTCAGAGCCTGTATTGGAATGGAA
ORP1-K555A-R	TTCCATTCCAATACAGGCTCTGAGGATGCTCCA
ORP1-R554A-K555A-F	TGGAGCATCCTCGCCGCCTGTATTGGAATG
ORP1-R554A-K555A-R	CATTCCAATACAGGCGGCGAGGATGCTCCA
ORP1-I557D-F	ATCCTCAGAAAATGTGACGGAATGGAACTATCC
ORP1-I557D-R	GGATAGTTCCATTCCGTCACATTTTCTGAGGAT
ORP1-K563A-F	GGAATGGAACTATCCGCCATCACGATGCCAGTT
ORP1-K563A-R	AACTGGCATCGTGATGGCGGATAGTTCCATTCC
ORP1-M566D-F	CTATCCAAGATCACGGACCCAGTTATATTTAAT
ORP1-Lid&N_TER loop Del.-F	GGCATCAAGAAACACCCAGTTATATTTAAT
ORP1-Lid&N_TER loop Del.-R	ATTAAATATAACTGGGTGTTTCTTGATGCC
ORP1-M566D-R	ATTAAATATAACTGGGTCCGTGATCTTGGATAG
ORP1-F570A-F	ACGATGCCAGTTATAGCCAATGAGCCTCTGAGC
ORP1-F570A-R	GCTCAGAGGCTCATTGGCTATAACTGGCATCGT
ORP1-Y583A-F	CAGCGCCTAACTGAAGCCATGGAGCATACTTAC
ORP1-Y583A-R	GTAAGTATGCTCCATGGCTTCAGTTAGGCGCTG
ORP1-P624A-F	CGGACTGGAAAAGCCTTCAACCCACTG
ORP1-P624A-R	CAGTGGGTTGAAGGCTTTTCCAGTCCG
ORP1-H651A-H652A-F	TCCGAACAGGTCAGCGCCGCACCACCAATCAGTGCA
ORP1-H651A-H652A-R	TGCACTGATTGGTGGTGCGGCGCTGACCTGTTCGGA
ORP1-Y674A-F	TTTCATGGCTCTATCGCCCCCAAACTGAAATTC
ORP1-Y674A-R	GAATTTCAGTTTGGGGGCGATAGAGCCATGAAA
ORP1-K676A-F	GGCTCTATCTATCCCGCGCTGAAATTCTGGGGG
ORP1-K676A-R	CCCCCAGAATTTCAGCGCGGGATAGATAGAGCC
ORP1-L677A-F	TCTATCTATCCCAAAGCCAAATTCTGGGGGAAG
ORP1-L677A-R	CTTCCCCCAGAATTTGGCTTTGGGATAGATAGA
ORP1-K678A-F	ATCTATCCCAAACTGGCGTTCTGGGGGAAGAGT

ORP1-K678A-R	ACTCTTCCCCCAGAACGCCAGTTTGGGATAGAT
ORP1-P688A-F	GTAGAAGCAGAAGCCAAAGGAACCATC
ORP1-P688A-R	GATGGTTCCTTTGGCTTCTGCTTCTAC
ORP1-K689A-F	GTAGAAGCAGAACCCGCCGGAACCATCACCTTG
ORP1-K689A-R	CAAGGTGATGGTTCCGGCGGGTTCTGCTTCTAC
ORP1-V712D-F	AATCCCACCTGCTGTGACCATAATATCATTGTG
ORP1-V712D-R	CACAATGATATTATGGTCACAGCAGGTGGGATT
ORP1-K763A-K765A-K766A-K767A-F (4K/4A)	TACATTCAAGATGCAAGCGCAGCAGCACTCTGTGCCCTC
ORP1-K763A-K765A-K766A-K767A-R (4K/4A)	GAGGGCACAGAGTGCTGCTGCGCTTGCATCTTGAATGTA
ORP1-K791A-K792A-F (2K/2A)	TTTGACGCTTACGCAGCAAATGATAAGAAA
ORP1-K791A-K792A-R (2K/2A)	TTTCTTATCATTTGCTGCGTAAGCGTCAAA
ORP1-K893A-F	CAAGCTAGTGAAGAAGCGAAACGACTTGAGGAA
ORP1-K893A-R	TTCCTCAAGTCGTTTCGCTTCTTCACTAGCTTG
ORP1-E897A-F	GAAAAAAAACGACTTGCGGAAAAACAAAGAGCA
ORP1-E897A-R	TGCTCTTTGTTTTTCCGCAAGTCGTTTTTTTTC
ORP1-R901A-F	CTTGAGGAAAAACAAGCGGCAGCCCGCAAAAAC
ORP1-R901A-R	GTTTTTGCGGGCTGCCGCTTGTTTTTCCTCAAG
ORP1-K905A-F	GCAGCCCGCGCCAACAGGTCC
ORP1-K905A-R	GGACCTGTTGGCGCGGGCTGC
ORP1-K909A-F	CGCAAAAACAGGTCCGCCTCAGAAGAGGACTGG
ORP1-K909A-R	CCAGTCCTCTTCTGAGGCGGACCTGTTTTTGCG
