# Supplementary material for: Allosteric enhancement of ORP1-mediated cholesterol transport by PI(4,5)P2/PI(3,4)P2
Source: Nat Commun. 2019 Feb 19;10:829. doi: 10.1038/s41467-019-08791-0 (PMC6381110; doi:10.1038/s41467-019-08791-0)
Supplement: Supplementary file 3 — Description of Additional Supplementary Files [file 41467_2019_8791_MOESM3_ESM.docx]

**Title:** Supplementary Movie 1
**Description:** MD simulations of the monomeric ORP1-ORD-cholesterol complex. The ORP1-ORD is shown in cartoon with the N-terminal loop in blue, lid in magenta and core in yellow. The cholesterol ligand is highlighted as green stick.
