# Supplementary material for: Allosteric enhancement of ORP1-mediated cholesterol transport by PI(4,5)P2/PI(3,4)P2
Source: Nat Commun. 2019 Feb 19;10:829. doi: 10.1038/s41467-019-08791-0 (PMC6381110; doi:10.1038/s41467-019-08791-0)
Supplement: Supplementary file 5 — Reporting Summary [file 41467_2019_8791_MOESM5_ESM.pdf]

nature research

Corresponding author(s): Jia-Wei Wu & Hongyuan Yang

Reporting Summary

Nature Research wishes to improve the reproducibility of the work that we publish. This form provides structure for consistency and transparency in reporting. For further information on Nature Research policies, see Authors & Referees and the Editorial Policy Checklist.

Statistical parameters

When statistical analyses are reported, confirm that the following items are present in the relevant location (e.g. figure legend, table legend, main text, or Methods section).

n/a	Confirmed	
☐	☒	The exact sample size (n) for each experimental group/condition, given as a discrete number and unit of measurement
☐	☒	An indication of whether measurements were taken from distinct samples or whether the same sample was measured repeatedly
☐	☒	The statistical test(s) used AND whether they are one- or two-sided<br>*Only common tests should be described solely by name; describe more complex techniques in the Methods section.*
☐	☒	A description of all covariates tested
☐	☒	A description of any assumptions or corrections, such as tests of normality and adjustment for multiple comparisons
☐	☒	A full description of the statistics including central tendency (e.g. means) or other basic estimates (e.g. regression coefficient) AND variation (e.g. standard deviation) or associated estimates of uncertainty (e.g. confidence intervals)
☐	☒	For null hypothesis testing, the test statistic (e.g. F, t, r) with confidence intervals, effect sizes, degrees of freedom and P value noted<br>*Give P values as exact values whenever suitable.*
☒	☐	For Bayesian analysis, information on the choice of priors and Markov chain Monte Carlo settings
☒	☐	For hierarchical and complex designs, identification of the appropriate level for tests and full reporting of outcomes
☒	☐	Estimates of effect sizes (e.g. Cohen's d, Pearson's r), indicating how they were calculated
☐	☒	Clearly defined error bars<br>*State explicitly what error bars represent (e.g. SD, SE, CI)*

Our web collection on statistics for biologists may be useful.

Software and code

Policy information about availability of computer code

Data collection	Blue-ice for synchrotron x-ray data collection.
Data analysis	HKL2000, Phaser, Coot, Phenix 1.13, PyMOL, VMD1.9.3, MODELLER9v10, NAMD 2.9, CHARMM36

For manuscripts utilizing custom algorithms or software that are central to the research but not yet described in published literature, software must be made available to editors/reviewers upon request. We strongly encourage code deposition in a community repository (e.g. GitHub). See the Nature Research guidelines for submitting code & software for further information.

Data

Policy information about availability of data

All manuscripts must include a data availability statement. This statement should provide the following information, where applicable:

- Accession codes, unique identifiers, or web links for publicly available datasets
- A list of figures that have associated raw data
- A description of any restrictions on data availability

The coordinates and structure factors have been deposited in the Protein Data Bank with accession codes 5ZM5 and 5ZM7 for ORP1-ORD in complex with cholesterol and 5ZM6 for ORP1-ORD in complex with PI(4,5)P2.

The source data underlying Figs 1b-d, 2b, 2d-k, 3b-d, 5b, 6a-b, 7a, 7c-d, 8a-b, 8f-g, 9b and Supplementary Figs 2a-b, 3b, 4a-b, 4e, 6a, 7, 8b, 9b-c, 10b, 11a-b, 12a-b are provided as a Source Data file.

Field-specific reporting

Please select the best fit for your research. If you are not sure, read the appropriate sections before making your selection.

☒ Life sciences ☐ Behavioural & social sciences ☐ Ecological, evolutionary & environmental sciences

For a reference copy of the document with all sections, see nature.com/authors/policies/ReportingSummary-flat.pdf

Life sciences study design

All studies must disclose on these points even when the disclosure is negative.

Sample size	No statistical approach was used to predetermine sample size. The determined sample size was adequate as the differences between experimental groups was significant and reproducible.
Data exclusions	No data were excluded from the analyses.
Replication	All attempts at replication were successful.
Randomization	This is not relevant to our study, because no grouping was needed.
Blinding	This is not relevant to our study.

Reporting for specific materials, systems and methods

Materials & experimental systems

n/a	Involved in the study	
☒	☐	Unique biological materials
☐	☒	Antibodies
☐	☒	Eukaryotic cell lines
☒	☐	Palaeontology
☒	☐	Animals and other organisms
☒	☐	Human research participants

Methods

n/a	Involved in the study	
☒	☐	ChIP-seq
☒	☐	Flow cytometry
☒	☐	MRI-based neuroimaging

Antibodies

Antibodies used	Anti-ORP1, Abcam, Cat#ab131165; Anti-p70 S6 Kinase, Cell Signaling, Cat#9202; Anti-phospho-p70 S6 Kinase (T389), Cell Signaling, Cat#9205; Anti-GAPDH (14C10), Cell Signaling, Cat#2118; p-T389 S6K (CST, catalog no. 9205); HA-Tag (CST, catalog no. 3724); and mouse monoclonal to GFP (Santa Cruz Biotechnology, catalog no. sc9996); purified PtdIns(3,4)P2 (Echelon Biosciences, catalog no. Z-P034).
Validation	Antibody validations were performed by antibody suppliers per quality assurance literature provided by each supplier.

Eukaryotic cell lines

Policy information about cell lines

Cell line source(s)	HeLa cells were acquired from ATCC.
Authentication	HeLa cells acquired from ATCC and no additional authentication was performed.
Mycoplasma contamination	All cell lines tested were negative for mycoplasma contamination.
Commonly misidentified lines (See ICLAC register)	No commonly misidentified lines were used in the study
